# Kivalliq Inuit women travelling to Manitoba for birthing: findings from the Qanuinngitsiarutiksait study

**DOI:** 10.1186/s12884-022-05214-9

**Published:** 2022-11-23

**Authors:** Josée G. Lavoie, Wayne Clark, Leah McDonnell, Nathan Nickel, Rachel Dutton, Janet Kanayok, Jack Anawak, Caroline Anawak, Levinia Brown, Grace Voisey Clark, Maata Evaluardjuk-Palmer, Frederick Ford, Melinda Fowler-Woods, Sabrina Wong, Julianne Sanguins, Alan Katz

**Affiliations:** 1 University of Manitoba, Winnipeg, MB Canada; 2 University of Alberta, Edmonton, AB Canada; 3 Manitoba Inuit Association, Winnipeg, MB Canada; 4 Isumataq Sivuliuqti, Iqaluit, Canada; 5 University of British Columbia, Vancouver, BC Canada; 6 Manitoba Metis Federation, Winnipeg, MB Canada

**Keywords:** Inuit health, Birthing, Pregnancy, Mothers

## Abstract

**Background:**

The Qanuinngitsiarutiksait study aimed to develop detailed profiles of Inuit health service utilization in Manitoba, by Inuit living in Manitoba (approximately 1,500) and by Inuit from the Kivalliq region of Nunavut who travel to Manitoba to access care not available in Nunavut (approximately 16,000 per year).

**Methods:**

We used health administrative data routinely collected in Manitoba for all services provided and developed an algorithm to identify Inuit in the dataset. This paper focused on health services used by Inuit from the Kivalliq for prenatal care and birthing.

**Results:**

Our study found that approximately 80 percent of births to women from the Kivalliq region occur in Manitoba, primarily in Winnipeg. When perinatal care and birthing are combined, they constitute one third of all consults happening by Kivalliq residents in Manitoba. For scale, hospitalizations for childbirths to Kivalliq women about to only 5 percent of all childbirth-related hospitalizations in Manitoba.

**Conclusions:**

The practice of evacuating women from the Kivalliq for perinatal care and birthing is rooted in colonialism, rationalized as ensuring that women whose pregnancy is at high risk have access to specialized care not available in Nunavut. While defendable, this practice is costly, and does not provide Inuit women a choice as to where to birth. Attempts at relocating birthing to the north have proven complex to operationalize. Given this, there is an urgent need to develop Inuit-centric and culturally appropriate perinatal and birthing care in Manitoba.

## Introduction

As a result of colonialism, Inuit culture has faced tremendous pressures in the past decades. Social policies aimed at times at fostering “modernity” among Inuit (i.e., educational policies, social welfare), and at times at extending some of the benefits of Canadian society (i.e., healthcare) have deliberately or inadvertently undermined Inuit culture, social fabric, and economy [1–3]. The following quote epitomizes the impact policies had on birthing:

In 1945 an Inuit woman gave birth to a healthy daughter in a tent on Inuit land in the Canadian Arctic. She was aided by an [I]ndigenous midwife and surrounded by her mother and other community women. In 1966, that daughter gave birth in her Inuit community with the prenatal, birthing, and postnatal care of a [western-trained] midwife. In 1986, that granddaughter, experiencing a low-risk pregnancy, was flown to a non-Indigenous southern hospital hundreds of miles away at 38 weeks gestation to deliver her baby ([4], p. 1).

The colonialization of birthing was operationalized through its medicalization [5]. Intents were framed as benevolent: as healthcare became more and more specialized, birthing in a hospital was framed to minimize clinical risks [6–9]. Cultural, emotional, economic and societal risks were not considered by policy makers [10]. Neither was the preference of Inuit women and their family.

In past decades, pressures have been mounting to bring back birthing to the north. This follows international trends for *birthing in country or place-based birthing* brought on by Indigenous communities [11, 12]. The community of Rankin Inlet is a central hub for the Kivalliq, and has had a birthing centre since 1993. Despite this, an important number of births still happen in Winnipeg, Manitoba.

This paper takes a closer look at trends in birthing locations for Inuit from the Kivalliq region of Nunavut. We present findings from the Qanuinngitsiarutiksait study, which was developed to determine user health care profiles for Inuit who travel from the Kivalliq region to access health care services, as well as Inuit who live in Manitoba. Qanuinngitsiarutiksait is the first and only study to provide a baseline of health care trends for Kivalliq residents accessing services in Manitoba and Inuit living in Manitoba.

Nunavut manages a very complex healthcare system. Nunavut communities are served by health centres where the bulk of care is provided by nurses working with an expanded scope of practice. Some larger communities, including Rankin Inlet, benefit from resident family physicians. Most communities however access care provided by a visiting family physician and some specialists a few days per month [13]. When this level of care is insufficient, Inuit must travel to larger provincial points of care. Inuit from the western part of Nunavut generally access such services in Edmonton Alberta. Inuit from the eastern part of Nunavut travel to Ottawa Ontario. For Inuit from the Kivalliq region, care is accessed in Winnipeg, Manitoba [14]. Services provided outside of Nunavut are billed back to Nunavut. Marchildon estimated that nearly 40 percent (2009 data) of all Nunavut healthcare expenditures ($263 M in 2009) is for care provided outside of the territory. In addition, nearly 18 percent of Nunavut’s health expenditures are for medical travel, either within the territory or to provincial points of care [13]. The creation of this system was led by the federal government from the 1950’s onward, without input from Inuit.

The adoption of technologies to measure obstetrical risks in the 1970’s was used as the justification for increased encroachment by the federal government on birthing. This encroachment was framed as a moral obligation to extend biomedical birthing care to all Inuit [15]. At the same time, changes in immigration policies, motivated largely by a shift led by the medical profession towards the medicalization of birth [16], drastically reduced the number of midwives that could be recruited from abroad [7]. By 1980, nearly all pregnant women from the north were being evaluated to a southern point of care for birthing, and gave birth under the supervision of a medical professional. In the Kivalliq region, this became policy in 1982, further undermining Inuit midwifery knowledge transmission and naming practices at birth (bestowed by grandparents and the midwife or *ikajurti*) [5, 17–20]. This policy is also linked to family stresses as women travel, until recently without their partner [21], to a southern locale at 36 weeks of gestation, to await their delivery.

Continuous attempts at expanding the care accessible in Nunavut have been hampered by a number of factors, including provider turnover [22], diseconomies of scale that makes the expansion of local services unrealistic given volume, and local workloads which can expand exponentially as a result of an outbreak, attrition or an accident [23]. Attempts at repatriating birthing to the Kivalliq regions came to the foreground in the mid 1980’s [17]. It was this activism that resulted in the opening of the Rankin Inlet Birthing Centre in 1993. Douglas reports that between 1993 and 2005, the centre supported 238 out of 506 births (47 percent). Of those who were evacuated, some were already in labour (10 people or 2 percent) while 24 (5 percent) were evacuated on the post-natal period. Douglas attributes the sizeable number of women still being evacuated for birth to restrictive risk-scoring methods and chronic staff shortages [24]. The Government of Nunavut’s maternal and newborn strategy notes the following indicators (Table 1).

**Table 1:** Health Status Measures [20, 2004 figures]

	**Canada**	**Nunavut**
Pre-term births (less than 37 weeks)	8%	12%
Rate of infant death (per 1000 live births)	5	16
Rate of neonatal death (per 1000 live births)	4	9
Rate of post neonatal death (per 1000 live births)	1	7
Rate of neonatal hospital readmission	3.5%	5.5%

The notion of risk, to the mother and baby, can explain in part the push for women to deliver in tertiary care centres. Predominantly biomedical constructs of risk, and associated legal risks to the provider and employer, are reductionists, and ignore cultural, social and personal risks to Inuit families [6], and Inuit’s own conceptualizations of risk [21, 25, 26]. Further, it is unclear how evacuating women to urban locales might mitigate risks associated with food insecurity [27], crowded housing [28], poverty and other systemic risk factors resulting from colonial encroachment on Inuit lives.

## Methods

### Participants

This study reports on findings from the *Qanuinngitsiarutiksait: Developing Population-Based Health and Well-Being Strategies for Inuit in Manitoba*, which was undertaken in partnership with the Manitoba Inuit Association and six Inuit Elders from Manitoba and Nunavut. The word Qanuinngitsiarutiksait means tools for the well-being/safety of Inuit/people. This study wanted to document the experience and needs of Inuit accessing health and other services in Manitoba, and develop strategies to enhance these experiences and facilitate transitions to and from Nunavut. Specific objectives included, 1) developing detailed profiles of Inuit accessing services including length of residence (permanent, short-term, or long-term relocation), types of services accessed, unmet needs, costs and challenges associated with relocating to and accessing services in Manitoba; and 2) exploring solution-oriented options with impacted families, the Manitoba Inuit Association, and allied health agencies presently serving Inuit in Manitoba.

We identified Inuit from the Kivalliq accessing services in Manitoba through their use of their Nunavut Healthcare Number (NHCN) card. This number is routinely recorded at the point of care for the purpose of Manitoba billing back services to Nunavut. Manitoba Health added a flag to this dataset, anonymized it and transferred it to the Manitoba Centre for Health Policy (MCHP). To determine the denominator, we used Statistics Canada Kivalliq populations, and inferred population figures in between census years.

Once our cohort was created, we linked these cohorts to administrative data housed in the Population Health Research Data Repository at MCHP. We were able to link this cohort to a long list of administrative datasets, including hospital separation abstracts; vital statistics; admission, discharge and transfer, E-triage; hospital abstracts; Manitoba Health Insurance Registry; and Medical Services. Our data linkage spanned 2001 to 2016.

The study received ethics approval from the University of Manitoba Health Research Ethics Board and data access approval from the Government of Manitoba Health Information Privacy Committee.

In this paper, we report on rate of childbirth hospitalizations of residents of the Kivalliq region in Winnipeg to: i) small Manitoban communities (defined as rural communities less than 8000), ii) the Northern Regional Health Authority and Churchill and, iii) the rate of all Manitoba. We look at the top reasons for diagnoses in Manitoba hospitals for women from the Kivalliq region and identify trends related to childbirth hospitalizations and other conditions originating in the perinatal period. The Kivalliq region’s population is small. In order look at and analyze health care trends our study uses a 5-year rolling average (we average the rate for periods of 5 years). This rolling average gives a more accurate picture of health care trends and needs.

The main measures reported here are the rates of childbirth hospitalization in Winnipeg for Kivalliq residents, and trends for childbirth hospitalizations and conditions originating in perinatal period, which include anything to do with the mother or the baby can impact the health of the baby or fetus during the period before birth, and through the first 28 days after birth.

## Results

**Fig. 1:**
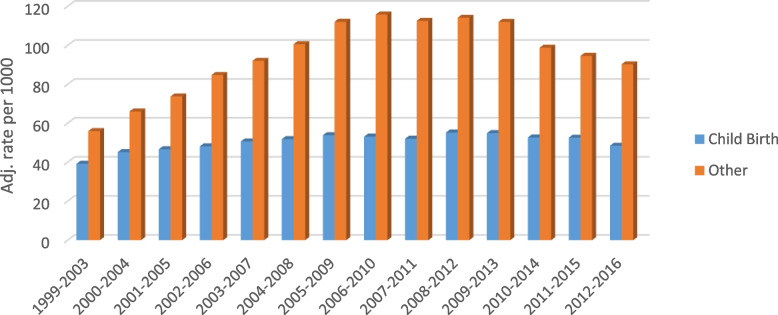
Adjusted rate per 1000 of Manitoba-based Childbirth vs Other Hospitalization, Kivalliq female residents, by 5 year rolling average

The Government of Nunavut reported 289 births to Kivalliq residents in 2011 [29]. Our data shows an average of 232 births to Kivalliq women occurring in Manitoba during the same period (80 percent). We attribute the discrepancy to birth that occurred in Nunavut, either at the Rankin inlet Health Centre, or communities when an evacuation did not occur in time. Some women may also elect to travel to Iqaluit for birthing. Figure 1 shows that 70 percent of all hospitalizations for women from the Kivalliq region in 1999–2003 and 54 percent of all hospitalizations in 2012–16 were for childbirth.

**Fig. 2:**
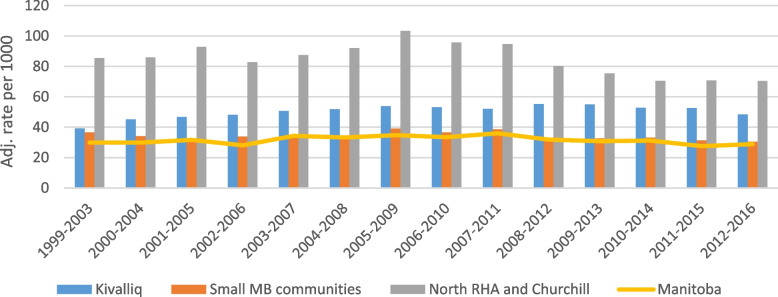
Adjusted rate of childbirth hospitalizations per 1,000 Kivalliq female residents, by 5 years roll-up

Figure 2 compares the adjusted rates of hospitalization for childbirth for female residents of the Kivalliq, small Manitoba communities, female residents from the northern regional authority and all Manitoba.

**Fig. 3:**
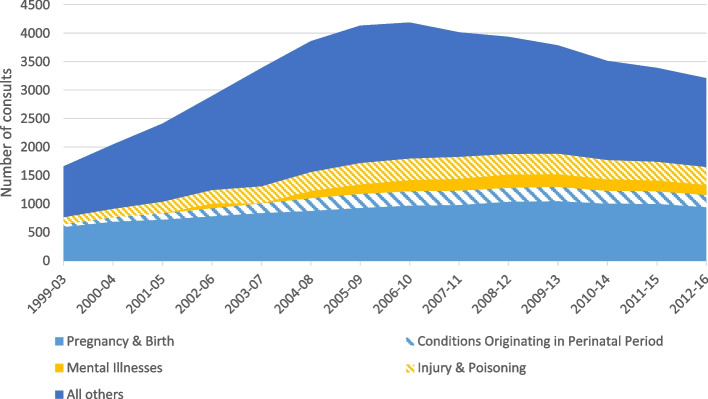
Number of Kivalliq Women's consults for birthing and peritanal conditions in Manitoba, compared total consults

For scale, the number of childbirth-related hospitalization in Manitoba to women from the Kivalliq (representing 80 percent of all births for women from the Kivalliq) averaged 952 per year, compared to 1,571 for Manitoba residents of small communities, 2,484 for residents of the northern Regional Health Authority and Churchill, and 17,917 for all of Manitoba (which includes small communities and the northern RHA and Churchill). So overall, while adjusted rates are much higher, hospitalizations for childbirth for Kivalliq residents account for approximately 5 percent of Manitoba hospitalizations for childbirth. Figure 3 shows that pregnancy, childbirth and conditions developing in the perinatal period account consistently for a third of all reasons why women from the Kivalliq region access care in Manitoba.

## Discussion

The Qanuinngitsiarutiksait study is a first attempt at documenting how and why Inuit access health services in Manitoba. The attempt breaks new methodological ground; still we note some limitations. *First*, we are aware that the population of Nunavut is 84.9 percent Inuit. This goes up to 91.1 percent for the Kivalliq region [30]. Thus, assuming that all Kivalliq residents utilize Manitoba Health services equally, we could assume at nearly 1 consult for every 10 is from a non-Inuit person. Yet, we also know that a majority of non-Inuit residents of Nunavut moved to Nunavut for employment, are generally older and likely to have a lower birth rate than Inuit. Consequently, we anticipate that the number of consults and hospitalizations by non-Inuit would be considerably less than 1 in 10.

*Second*, the Government of Nunavut reported 289 births to Kivalliq residents in 2011 [23]. Our data shows an average of 232 births to Kivalliq women occurring in Manitoba during the same period. The discrepancy between these numbers might represent the number of births occurring in Nunavut communities [24]. For example, a news article published in 2014 reported approximately 50 births per year at the Rankin Inlet Birthing Centre ([31], statistics from official sources could not be located). In addition, births do occur in communities’ health centres when women present in labour and/or when transportation is not possible because of weather conditions and other factors.

*Third,* we did not attempt to quantify the proportion of high-risk births occurring in Manitoba. While assessing risk from a strictly clinical perspective is possible, we feel that a more culturally appropriate perspective should be used. The development of a culturally grounded algorithm for identifying high risk pregnancies in administrative data will require additional work.

Despite these limitations, our findings provide a lens into the number of Inuit births occurring in Manitoba. Our data shows that an average of 262 births still occur in Winnipeg (80 percent). At an estimated $14,000/births ([32], this figure assumes that transportation was through a scheduled flight and not an emergency, which would double the cost) and as a point of reference, this practice costs Nunavut an estimated $3.7 M per year. While sizable for Nunavut, Manitoba sees nearly 18,000 hospitalizations related to childbirth every year, compare to less than 1,000 for Kivalliq residents. Despite the best of intentions, it is doubtful that service providers can gain sufficient experience from their Inuit patient contacts to ensure that they deliver a culturally appropriate and Inuit-centric approach. At 5 percent of all childbirth-related hospitalizations, awareness raising is essential to ensure that Inuit can access culturally appropriate care in Winnipeg’s hospital.

Despite aspirations to relocate care birthing to Nunavut, it is likely that the current trend will continue for years to come, partially because of staff shortage in Nunavut. Over the past decades, on average, half of Nunavut’s health professional positions have been vacant at any given time [33]. The turnover of staff trained and recruited from the southern part of Canada is associated with a myriad of factors including isolation, working with an expanded scope of practice in a more autonomous manner than what nurses are generally accustomed to leading to exhaustion for some, the high cost of living, lack of employment opportunities for life partners, etc. [22]. This has led to a reliance on short term contract nurses who by definition, may have limited knowledge of the community [23]. Pressures on the Rankin Inlet Birthing Centre recently resulted in its closure [34, 35] amidst concerns of marginalization and racism by Inuit midwives. Some births were rerouted to Iqaluit as a result.

Our results justify the need to develop Inuit-centric programming related to prenatal care and culturally grounded approaches to birthing in Winnipeg. Since all care provided in Manitoba is paid for by the government of Nunavut, it seems reasonable that Nunavut should be leading discussions with Manitoba to ensure that culturally appropriate prenatal, birthing and post-natal care is available to Inuit women birthing in Manitoba.

## Conclusions

The focus on repatriating birth to the north has possibly *allowed* Manitoba and other southern points of care to continue to offer culturally generic care to Inuit: at the time of writing, access to Inuit-centric and cultural safe birthing remains aspirational in Manitoba and in other southern locales: despite over five decades of Inuit birthing in those locales, and increased attention to the importance of culturally safe care [36, 37], Inuit remain largely invisible.

Our findings highlight the need to urgently develop Inuit-centric prenatal and birthing services, to ensure access to safe, culturally appropriate, and responsive services. In addition, Inuit women awaiting birthing might require socio-cultural supports such as opportunities to engage in Inuit cultural events, access to traditional food, and other supports as well. Such services, despite decades of Inuit women accessing birthing care in Manitoba, have yet to emerge. Developing such services in partnership with Inuit women is essential to improving cultural safety, aligned with the Government of Manitoba’s commitment to Reconciliation [38] and is a stepping stone to improving birth outcomes for Inuit.

## Data Availability

The datasets generated and analyzed for this study are protected under the privacy policies of the Manitoba Centre for Health Policy, in compliance with Canadian and provincial privacy legislation. Access is subject to ethics approval at the University of Manitoba, approval from the Government of Manitoba’s Health Information Privacy Committee, and the support of the Manitoba Inuit Association, in compliance with the Tri-Council Guidelines for ethical research with Indigenous Peoples (https://ethics.gc.ca/eng/policy-politique_tcps2-eptc2_2018.html). Access to the data is regulated by these processes, and may only be accessed by complying with these processes.

## References

[1] Pollock NJ, Healey GK, Jong M, Valcour JE, Mulay S (2018). Tracking progress in suicide prevention in Indigenous communities: a challenge for public health surveillance in Canada. BMC Public Health.

[2] Statistics Canada (2012). Inuit health: Selected findings from the 2012 Aboriginal Peoples Survey.

[3] Inuit Tapiriit Kanatami (2014). Social determinants of Inuit health in Canada.

[4] Dreaddy K. Indigenous obstetric evacuation: harmful but risky to resist. Impact Ethics. 2019. Dec 19, 2019. https://impactethics.ca/2019/12/19/indigenous-obstetric-evacuation-harmful-but-risky-to-resist/. Accessed 30 Feb 2022.

[5] Kaufert PA, O'Neil JD (1990). Cooptation and control: the reconstruction of Inuit birth. Med Anthropol Q.

[6] O'Neil JD, Kaufert PA, Brown P, Voisey E, Moffatt MM, Postl B (1988). Inuit concerns about obstetric policy in the Keewatin region N.W.T. Arctic Med Res.

[7] Douglas VK (2006). Childbirth among the Canadian Inuit: a review of the clinical and cultural literature. Int J Circumpolar Health.

[8] Hobart CW (1975). Socioeconomic correlates of mortality and morbidity among Inuit infants. Arct Anthropol.

[9] Murdock AI (1979). Factors associated with high-risk pregnancies in Canadian Inuit. Can Med Assoc J.

[10] Wright A (2015). Role of the nurse in returing birth in the North. Rural Remote Health.

[11] Hickey S, Roe Y, Ireland S, Kildea S, Haora P, Gao Y (2021). A call for action that cannot go to voicemail: research activism to urgently improve Indigenous perinatal health and wellbeing. Women Birth.

[12] Brubacher LJ (2021). (Re)birthing systems in the Qikiqtaaluk Region of Nunavut: a place-based inquiry into Inuit Birthing, systems of care, and maternal health research.

[13] Marchildon GP, Torgenson R (2013). Nunavut: a health system profile.

[14] Clark W, Lavoie JG, McDonnell L, Nickel N, Anawak J, Brown L (2022). Trends in Inuit health services utilisation in Manitoba: findings from the Qanuinngitsiarutiksait study. Int J Circumpolar Health.

[15] O'Neil JD, Kaufert P, Ginsberg F, Rapp R (1997). Irniktakpunga!: sex determination and the Inuit struggle for birthing rights in Northern Canada. Conceiving the new world order: global and local intersections in the politics of reproduction.

[16] Lynch B (2005). Midwifery in the 21st century: the politics of economies, medicine, and health. J Midwifery Womens Health.

[17] Bornstein T (2020). Vol II: bringing birth home: an analysis of Inuit birthing practices and policies. McGill Journal of Global Health.

[18] O'Neil J, Kaufert P, Handwerker WP (1990). The politics of obstetric care: the Inuit experience. Births and power.

[19] Kaufert P, Moffatt M, O'Neil J, Postl B. The epidemiology of obstetric care in the Keewatin District: methodological issues. O'Neil J, Gilbert P, editors. Winnipeg: Winnipeg Northern Health Research Unit; 1990. p. 5–11.

[20] Baskett TF (1979). Obstetric care in the central canadian arctic. Obstet Gynecol Surv.

[21] Lee E, Gudmundson B, Lavoie JG. Returning childbirth to Inuit communities in the Canadian Arctic. Int J Circumpolar Health. 2022. Under review. 10.1080/22423982.2022.2071410 PMC9067959 35491889

[22] Cherba M, Healey Akearok GK, MacDonald WA (2019). Addressing provider turnover to improve health outcomes in Nunavut. CMAJ.

[23] McDonnell L, Lavoie JG, Healey G, Wong S, Goulet S, Clark W (2019). Non-clinical determinants of Medevacs in Nunavut: perspectives from northern health service providers and decision-makers. Int J Circumpolar Health.

[24] Douglas VK (2011). The Rankin Inlet Birthing Centre: community midwifery in the Inuit context. Int J Circumpolar Health.

[25] Qinuajuak L (1996). Inuit birth traditions. Midwifery Today Childbirth Educ.

[26] Qinuajuak L (1996). Birth is a normal part of life. Midwifery Today Childbirth Educ.

[27] St-Germain AF, Galloway T, Tarasuk V (2019). Food insecurity in Nunavut following the introduction of Nutrition North Canada. CMAJ.

[28] Riva M, Fletcher C, Dufresne P, Perreault K, Muckle G, Potvin L (2020). Relocating to a new or pre-existing social housing unit: significant health improvements for Inuit adults in Nunavik and Nunavut. Can J Public Health.

[29] Government of Nunavut. Community Statistics Nunavut 2016: Office of the Chief Medical Officer of Health, Technical Report. Nunavut DoHGo, editor. Iqaluit: Department of Health Government of Nunavut; 2016.

[30] Statistics Canada (2016). Profile by region and community, Kivalliq region.

[31] Rogers S. Nunavut’s oldest, newest birthing centres bring new life to Nunavut. Nunatsiaq News. 2014. 23 June 2014.

[32] May K. Birth in a Northern Nation. Winnipeg: Winnipeg Free Press; 2014.

[33] Government of Nunavut (2021). Inuit employment statistics.

[34] Rogers S (2020). Midwives depart, suspending prenatal services in Rankin Inlet. Nunatsiaq News.

[35] Tranter E. Inuit midwives say they left Nunavut centre after years of mistreatment. Winnipeg: The Canadian Press; 2021.

[36] Corcoran PM, Catling C, Homer CS (2017). Models of midwifery care for Indigenous women and babies: a meta-synthesis. Women Birth.

[37] Churchill ME, Smylie JK, Wolfe SH, Bourgeois C, Moeller H, Firestone M (2020). Conceptualising cultural safety at an Indigenous-focused midwifery practice in Toronto, Canada: qualitative interviews with Indigenous and non-Indigenous clients. BMJ Open.

[38] Government of Manitoba. Reconciliation strategy. Winnipeg: Indigenous Reconciliation and Northern Relations; n.d.

